# The published trend of studies on COVID-19 and dietary supplements: Bibliometric analysis

**DOI:** 10.3389/fimmu.2022.1065724

**Published:** 2022-11-16

**Authors:** Wenzhong Hu, Yun Xu

**Affiliations:** ^1^ Guang’anmen Hospital Southern District, China Academy of Chinese Medical Sciences, Beijing, China; ^2^ People’s Hospital of Beijing Daxing District, Capital Medical University, Beijing, China

**Keywords:** coronavirus disease 2019(COVID-19), vitamin D, bibliometric analysis, Citespace, vitamin C

## Abstract

**Background:**

There are no guidelines on dietary supplements for the prevention or treatment of COVID-19. Therefore, we would like to analyze and discuss the above confusion through the bibliometric analysis

**Methods:**

On 3 September 2022, we conducted a search of all relevant literature retrieved from the WOS core collection database from 2019 to 2022. CiteSpace software is used to build the visual co-occurrence network

**Results:**

In the study of “COVID-19 and Dietary Supplements”, the total of 170 authors published 855 articles in 451 journals. Several distinct core author groups were formed by Wang, Grant, Singh, Zhu, and other authors with numerous publications. The majority of the publications came from the Shahid Beheshti University of Medical Sciences. The United States of America had the highest number of publications. By analyzing keyword clusters, we found that the research focus was dietary supplements (vitamin D, vitamin K, vitamin C), mechanisms (ferritin, specialized pro-resolving mediators (SPMs), oxidative stress), research methods (clinical trials), and the prevention and treatment strategies (lockdown) of COVID-19

**Conclusions:**

vitamin D is the mainstream dietary supplement for COVID-19. There are still numerous controversies that deserve further discussion. Such as whether the use of vitamin D or TCM offers benefits, and whether the addition of dietary supplements during the lockdown measures can help prevent COVID-19 infection.

## Introduction

The SARS-CoV-2 virus is responsible for the infectious disease known as coronavirus disease 2019 (COVID-19) (1). According to data compiled by the World Health Organization (WHO), as of September 3, 2022, over 601 million confirmed cases of COVID-19 have resulted in more than 6.4 million deaths (2). Anyone can get sick with COVID-19 and become severely ill or die at any age. However, the elderly (3) and those with chronic diseases such as cardiovascular disease (4), diabetes (5), chronic respiratory disease, chronic kidney disease or cancer (6, 7) are more likely to develop serious illness. As we all understand it, an apple a day keeps the doctor away. Fruits contain a variety of nutrients, including vitamins, minerals, etc., that seem to be beneficial for us to fight against disease. Not only the nutrients in fruits, but also other dietary supplements are closely related to our lives. Micronutrients are essential for the normal functioning of the immune system and play a vital role in promoting health and nutritional health.In addition to vitamins and minerals, probiotics are among the dietary supplements that people frequently take. The regulation of the gut microbiota by probiotics has been suggested as a promising adjuvant approach for improving the health of patients with COVID-19 (8). However, in the context of the current COVID-19 pandemic, whether dietary supplements are still consistently recommended by scholars, whether they are beneficial to people in the prevention and treatment of COVID-19, and which dietary supplements are the focus of current research are topics of recent interest. This study is based on contributions made by other researchers for covid-19 and nutrition. For example, Muhammad Waseem Shah et al. interest on vitamin D and COVID-19 (9), Sa’ed H. Zyoud et al. focus on nutrition and COVID-19 (10). There are no guidelines on dietary supplements for the prevention or treatment of COVID-19. Therefore, we would like to analyze and discuss the above confusion through the bibliometric analysis, which can predict publication trends in a certain research field (11).

## Methods

### Data sources and retrieval strategies

**Figure 1:**
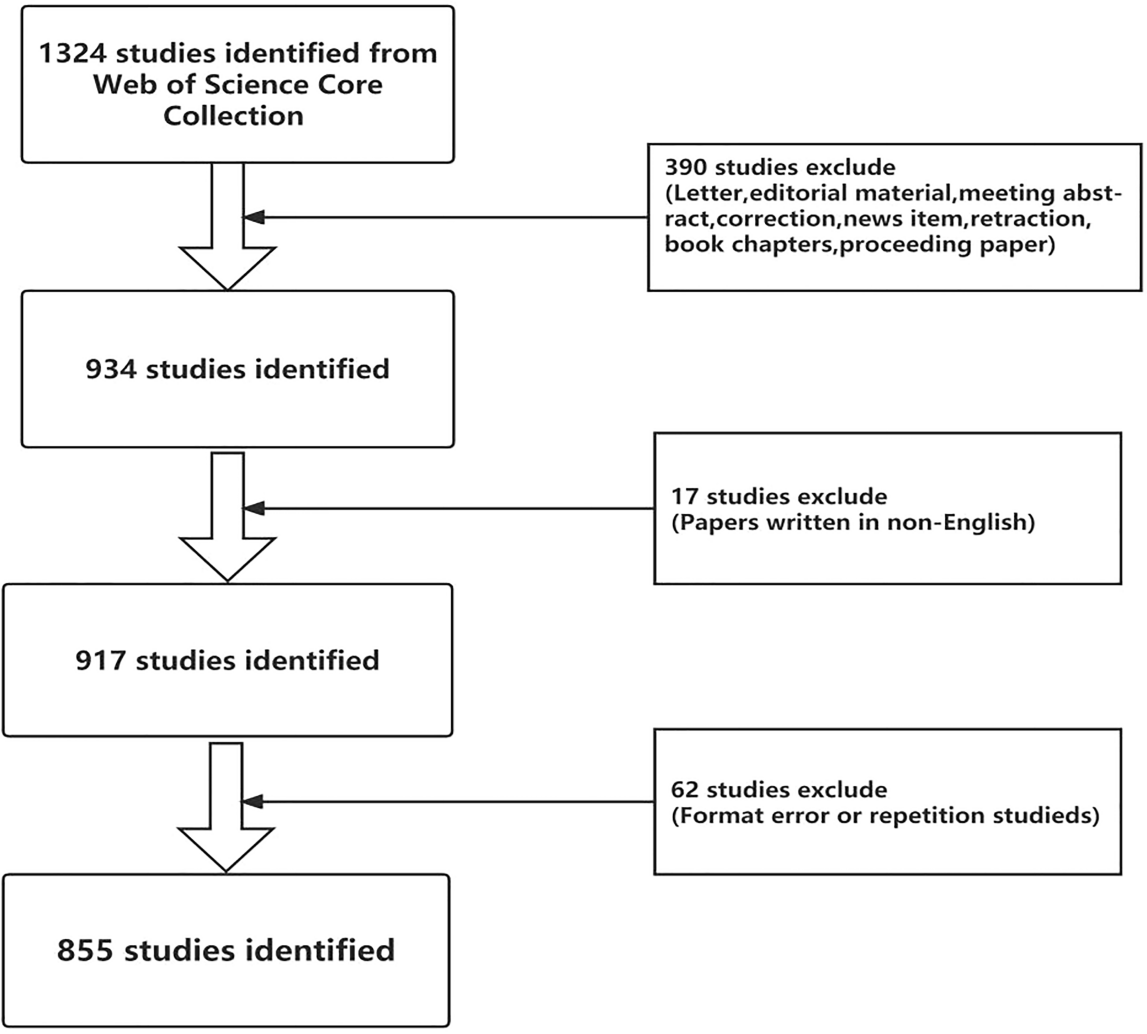
Detailed flowchart of search, screening on the Web of Science.

The Web of Science (WOS) is a comprehensive and multidisciplinary database, which has become the most adopted database for bibliometric analysis (12, 13). It provides general information about publications, including the author, the author’s affiliation, the publisher, etc. With citation reporting, we can quickly locate influential research and discover research hot spots for scholars and institutions around the world. In this way, we can better harness the true potential of this evolving discipline. On September 3, 2022, we used MeSH words to search all relevant literature or review articles from the WOS core collection database from 2019 to 2022. And we selected all the editions in the WOS core collection database. Two authors separately performed the literature search. The search formula is as follows:(TI= (Dietary Supplements) OR (Vitamin) OR (Mineral) OR (Zinc) OR (folic acid) OR (Probiotics) OR (fatty acids) OR (Co-enzyme Q10) OR (Iron)) AND (TI=(COVID 19) OR (2019 novel coronavirus) OR (coronavirus2019) OR (coronavirus disease 2019) OR (2019-novel CoV) OR (2019 ncov) OR (COVID 2019) OR (coronavirus 2019) OR (nCoV-2019) OR (nCoV2019) OR (2019-ncov) OR (COVID-19) OR (Severe acuterespiratory syndrome coronavirus 2) OR (SARS-CoV-2)). LANGUAGE: (English) AND DOCUMENT TYPES: (Article OR Review). Our search covered the period from December 1, 2019 to September 3, 2022. **Figure 1** displays comprehensive information regarding literature screening.

### Inclusion and exclusion criteria

By reading the titles, abstracts and keywords of the detected articles, the periodical articles with the theme of “COVID-19 and Dietary Supplements” were included. Articles with incomplete research information, conference articles, letter, editorial material, book chapters, news item, proceeding paper,retraction,and duplicate articles were excluded. To ensure the integrity and reliability of the study data, we placed the identification and elimination of duplicate studies as the final step in the screening. The studies were initially filtered by EndNote software and manually checked for duplicates, the rest were entered into CiteSpace and then automatically completed by the software’s “Remove Duplicate (WoS)” program. The identification of irrelevant studies were performed by two authors with medical backgrounds, who read the title, keywords and abstract to identify irrelevant literature.Two authors separately performed the studies. Any differences were resolved through secondary discussion. The results were discussed and decided after two authors independently read the full text.

### Analyzing tools and statistical methods

CiteSpace software is used to construct the visual co-occurrence network. CiteSpace is a web-based Java application for analyzing and visualizing co-citation networks (14).

We performed a comprehensive bibliometric analysis of the retrieved literature on COVID-19 and Dietary Supplements using CiteSpace 6.1.R2 (64-bit).We set the parameters in CiteSpace as follows: ① timespan = January 2019 to December 2022; ② the time slice = 1; ③ node type = author/institution/country/keyword/reference; ④ threshold selection criteria = the top 25 resultsfor each time slice. We set the other parameters by default.⑤ “Pathfinder” is selected as the cutting connection mode to simplify the network structure and highlight important features.

## Analysis results and visualization

As shown in **Figure 1**, we initially obtained 1324 studies by search terms. Secondly, filtering out 390 studies (including letters, editorial material, conference waivers, corrections, news entries, retractions, book chapters, dissertations), we got 934 studies (Article 625; Review Article 309). Then filtering out 17 studies that were not written in English, we got 917 studies. After loading the data into CiteSpace 6.1.R2 (64-bit) and running it, we filtered out 62 studies that had formatting errors or duplicates, and we ended up with 855 studies.

### Analysis of journals and co-cited journals

This study found 855 articles that were published in 451 journals. **Table 1** shows the top 9 journals in terms of published articles. The journals that had the most studies published on “COVID-19 and Dietary Supplements” were Nutrients (61,7.13%), followed by Journal of Medical Virology(13,1.52%),International Journal of Infectious Diseases(12,1.40%),Scientific Reports(12,1.40%),Frontiers in Immunology(11,1.29%),Frontiers in nutrition(11,1.29%),Frontiers in pharmacology(11,1.29%),International Journal of Environmental Research and Public Health(11,1.29%), and Nutrition(11,1.29%). The average impact factor, taken from the perspective of influence, was 8.371. The top 10 journals’ co-citation networks are shown in **Table 2**. There are 3,449 links and 694 nodes in this network. The most frequently cited journals were Nutrients (605,17.54%), followed by PLoS One (461,13.37%), Lancet (406,11.77%), JAMA-Journal of the American Medical Association (397,11.51%), and The New England journal of medicine (382,11.08%), with over 380 co-citation frequencies, accounting for more than 10%. The average impact factor was 73.169.

**Table 1:** Top 9 journals ranked by the number of publications.

Rank	Journal Names	Publications	% (N=855)	Impact factors
1	Nutrients	61	7.13	6.706
2	Journal of Medical Virology	13	1.52	20.693
3	International Journal of Infectious Diseases	12	1.40	12.074
4	Scientific Reports	12	1.40	4.996
5	Frontiers in Immunology	11	1.29	8.786
6	Frontiers in nutrition	11	1.29	6.59
7	Frontiers in pharmacology	11	1.29	5.988
8	International Journal of Environmental Research and Public Health	11	1.29	4.614
9	Nutrition	11	1.29	4.893

**Table 2:** Top 10 frequency cited journals.

Rank	Journal Names	Quantity	% (N=3,449)	Impact factors
1	Nutrients	605	17.54	6.706
2	PLoS One	461	13.37	3.752
3	Lancet	406	11.77	202.731
4	JAMA-Journal of the American Medical Association	397	11.51	157.335
5	The New England journal of medicine	382	11.08	176.079
6	BMJ-British Medical Journal	323	9.37	93.333
7	American Journal of Clinical Nutrition	319	9.25	8.472
8	Frontiers in Immunology	313	9.08	8.786
9	Scientific Reports	312	9.05	4.996
10	Nature	261	7.57	69.504

### Analysis of co-cited reference

**Figure 2:**
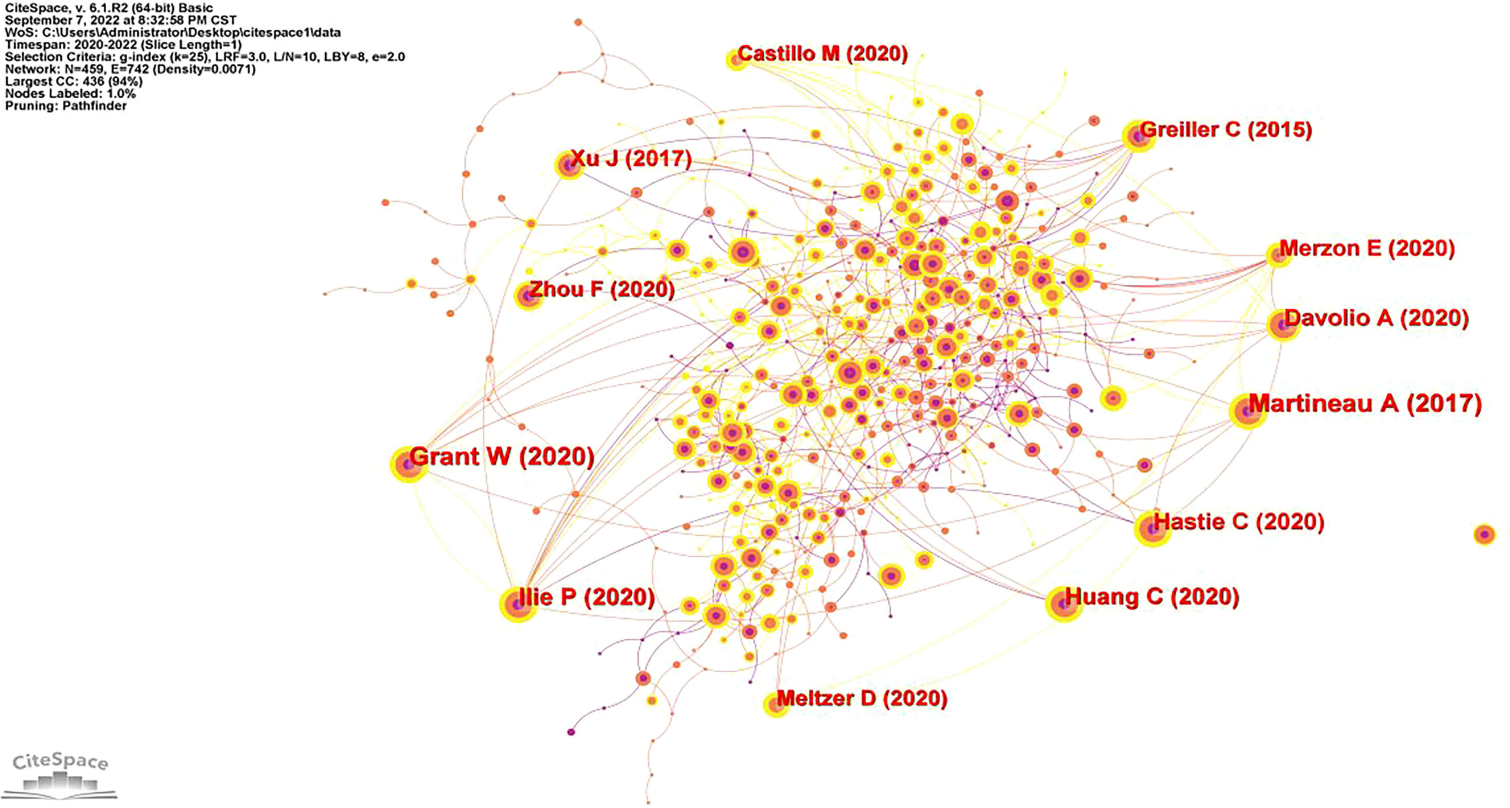
Map of co-cited references.

**Figure 2** depicts a map of the co-cited references. There were 742 connections and 459 nodes found. A cited reference is represented by each node. The number of co-cited references is represented by the size of the node. Co-citations are represented by the connection between nodes. A connection that is wider indicates a higher frequency of co-citations.

The top 5 co-cited references with frequency are presented in **Table 3**. The most co-cited article by Adrian R Martineau (2017), entitled “vitamin D supplementation to prevent acute respiratory tract infections: systematic review and meta-analysis of individual participant data” was published in BMJ-British Medical Journal (15). Then followed by William B Grant (2020), entitled “Evidence that vitamin D Supplementation Could Reduce Risk of Influenza and COVID-19 Infections and Deaths” was published in Nutrients (16); Petre Cristian Ilie (2020), entitled “The role of vitamin D in the prevention of coronavirus disease 2019 infection and mortality” was published in Aging Clinical and Experimental Research (17); Chaolin Huang (2020), entitled “Clinical features of patients infected with 2019 novel coronavirus in Wuhan, China” was published in Lancet (18) and Claire E Hastie (2020), entitled “vitamin D concentrations and COVID-19 infection in UK Biobank” was published in Diabetes and Metabolic Syndrome Clinical Research and Reviews (19). **Table 4** shows the top five co-cited references with the highest centrality. Petre Cristian Ilie was the top co-cited reference with the highest centrality (0.16), who published “The role of vitamin D in the prevention of coronavirus disease 2019 infection and mortality “ in the Journal of Aging Clinical and Experimental Research (20). And in this study, we found that the research contents of the top 4 articles with high centrality were related to vitamin D and respiratory diseases, such as COVID-19, influenza prevention or Therapy,respiratory virus infection and pneumocystis pneumonia.

**Table 3:** The top 5 frequency of co-cited reference.

Rank	Author	Title	Frequency	Publication year	Journal	Centrality
1	Adrian R Martineau	Vitamin D supplementation to prevent acute respiratory tract infections: systematic review and meta-analysis of individual participant data	194	2017	BMJ-British Medical Journal	0.03
2	William B Grant	Evidence that vitamin D Supplementation Could Reduce Risk of Influenza and COVID-19 Infections and Deaths	184	2020	Nutrients	0.08
3	Petre Cristian Ilie	The role of vitamin D in the prevention of coronavirus disease 2019 infection and mortality	154	2020	Aging Clinical and Experimental Research	0.16
4	Chaolin Huang	Clinical features of patients infected with 2019 novel coronavirus in Wuhan, China	135	2020	Lancet	0.13
5	Claire E Hastie	Vitamin D concentrations and COVID-19 infection in UK Biobank	109	2020	Diabetes and Metabolic Syndrome Clinical Research and Reviews	0.02

**Table 4:** The top 5 centrality of co-cited reference.

Rank	Author	Title	Frequency	Publication year	Journal	Centrality
1	Petre Cristian Ilie	The role of vitamin D in the prevention of coronavirus disease 2019 infection and mortality	154	2020	Aging Clinical and Experimental Research	0.16
2	Mihnea T Zdrenghea	Vitamin D modulation of innate immune responses to respiratory viral infections	42	2017	Reviews in Medical Virology	0.14
3	Beata M Gruber-Bzura	Vitamin D and Influenza-Prevention or Therapy?	43	2018	International Journal of Molecular Sciences	0.13
4	Guang-Sheng Lei	Mechanisms of Action of vitamin D as Supplemental Therapy for Pneumocystis Pneumonia	24	2017	Antimicrobial Agents and Chemotherapy	0.13
5	Diane Godeau	Return-to-work, disabilities and occupational health in the age of COVID-19	21	2020	Scand J Work Environ Health	0.13

### Analysis of authors

**Figure 3:**
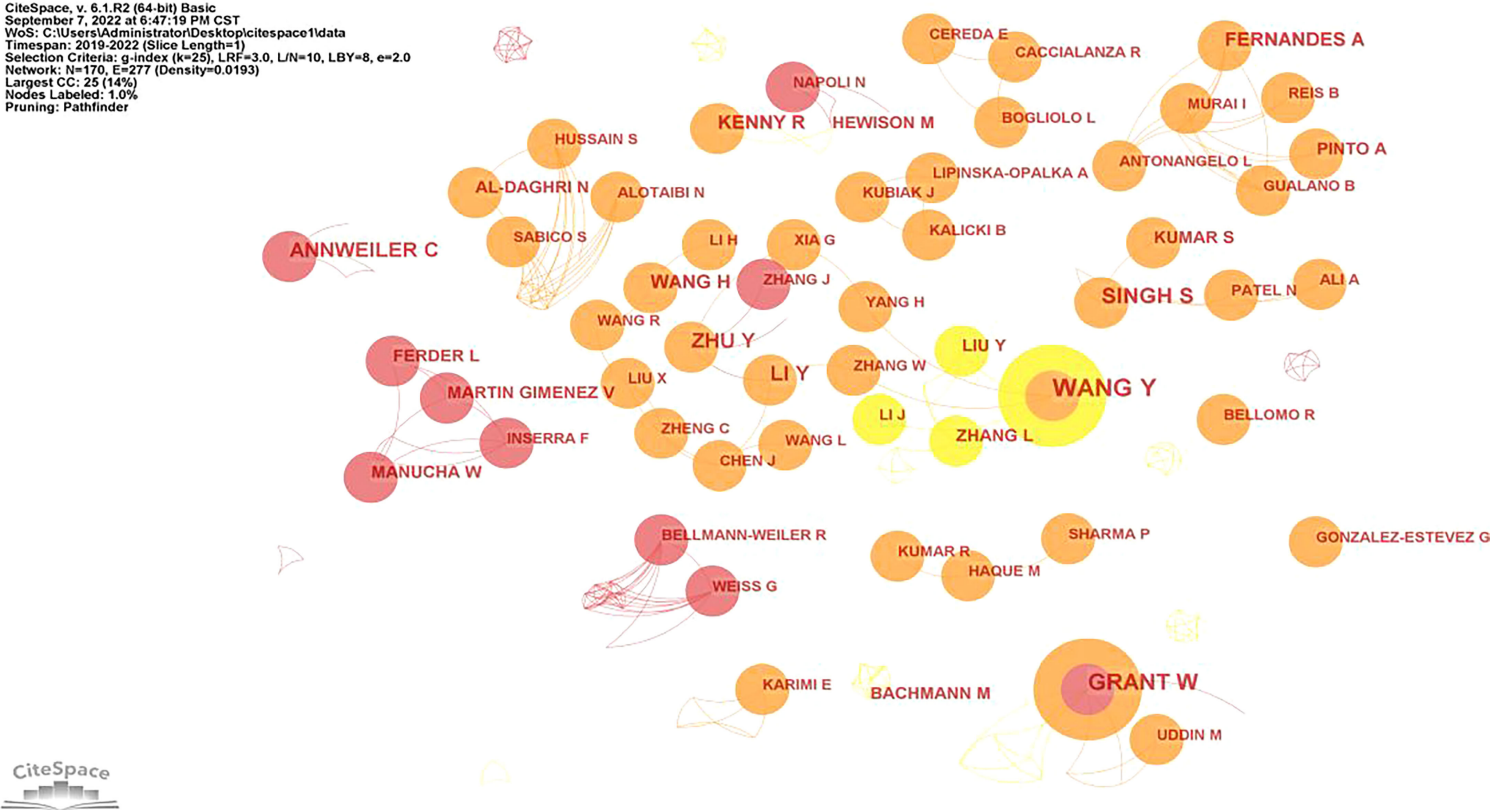
Network map showing authors’ collaborations in COVID-19 and Dietary Supplements research.

CiteSpace was used to investigate author cooperation networks in the literature, and it was discovered that 170 authors are participating in the production of publications linked to COVID-19 and Dietary Supplements research. We listed the top 10 authors by number of published works. See **Table 5** for details. Wang’s 10 publications make a significant contribution to the field, followed by Grant (8 publications), Singh (7 publications), Zhu (6 publications), Annweiler (6 publications), and Kershaw (6 publications). The collaboration between authors contains 170 nodes and 277 connections, with a network density of 0.0193. The number of published articles increases with node diameter. The fact that the nodes are connected suggests a joint effort by the authors. The network density indicates that the cooperation between authors is not close, but the cooperation between authors can be seen intuitively from the cooperation map, especially some core authors and team cooperation of the study. See **Figure 3** for details. Several distinct core author groups were formed by Wang, Grant, Singh, Zhu, and other authors with numerous publications. The core author group works closely together, but there are few connections between the various groups.

**Table 5:** Top 10 authors by number of published works.

Rank	Author	Publications	Citations	Rank	Author	Publications	Citations
1	Wang Y	10	59	6	Wang H	6	23
2	Grant W	8	812	7	Khan A	5	7
3	Singh S	7	54	8	Kenny R	5	56
4	Zhu Y	6	151	9	Holick M	5	205
5	Annweiler C	6	259	10	Fernandes A	5	224

### Analysis of institutions

**Figure 4:**
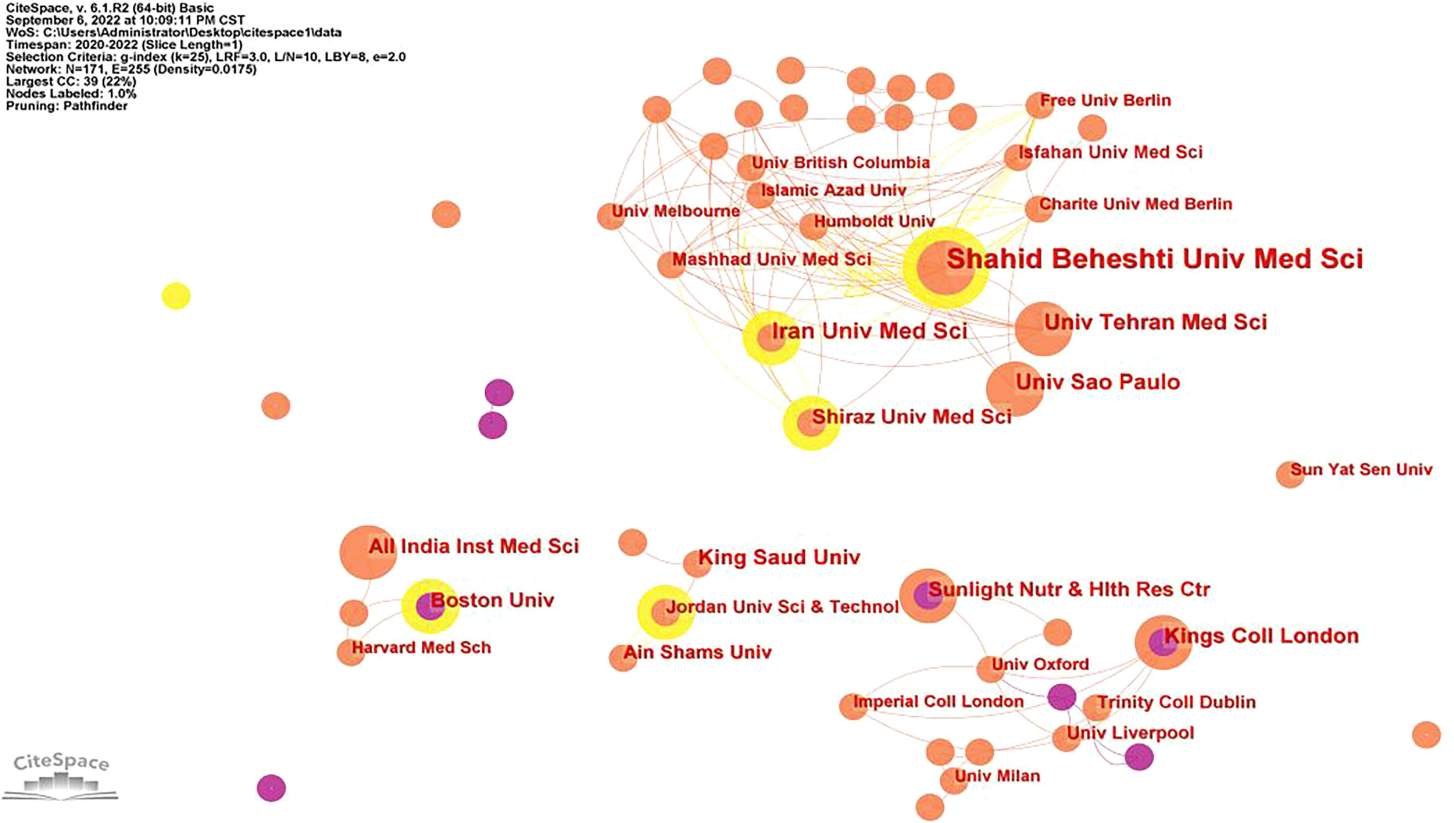
A network map showing institutional collaborations.

**Table 6** shows the top 10 research institutions. The majority of the publications came from the Shahid Beheshti University of Medical Sciences (22,2.57%), followed by the Iran University of Medical Sciences (11,1.29%), University of Sao Paulo (10,1.17%) and Tehran University of Medical Sciences (10,1.17%). An institutional collaboration map for the COVID-19 and Dietary Supplements research is depicted in **Figure 4**. This research was made possible by 171 institutions, and 255 connections were found. The number of publications is proportional to the size of each node, which represents an institution. It is obvious from the figure that four research groups have been formed in different research institutions. The largest research team is closely connected with Shahid Beheshti University of Medical Sciences as the center.

**Table 6:** Top 10 research institutions in the field.

Rank	Institution	Publications	% (N=855)	year	Country/region
1	Shahid Beheshti University of Medical Sciences	22	2.57	2020	Islamic Republic of Iran
2	Iran University of Medical Sciences	11	1.29	2021	Islamic Republic of Iran
3	University of Sao Paulo	10	1.17	2020	The Federative Republic of Brazil
4	Tehran University of Medical Sciences	10	1.17	2021	Islamic Republic of Iran
5	King Saud University	9	1.05	2020	Kingdom of Saudi Arabia
6	King’s College London	9	1.05	2020	The United Kingdom of Great Britain and Northern Ireland
7	All India Institute of Medical Sciences	8	0.94	2021	The Republic of India
8	Sunlight, Nutrition And Health Research Center	8	0.94	2020	The United States of America
9	Shiraz University of Medical Science	8	0.94	2021	Islamic Republic of Iran
10	Boston University	8	0.94	2020	The United States of America

### Analysis of countries/regions

**Figure 5:**
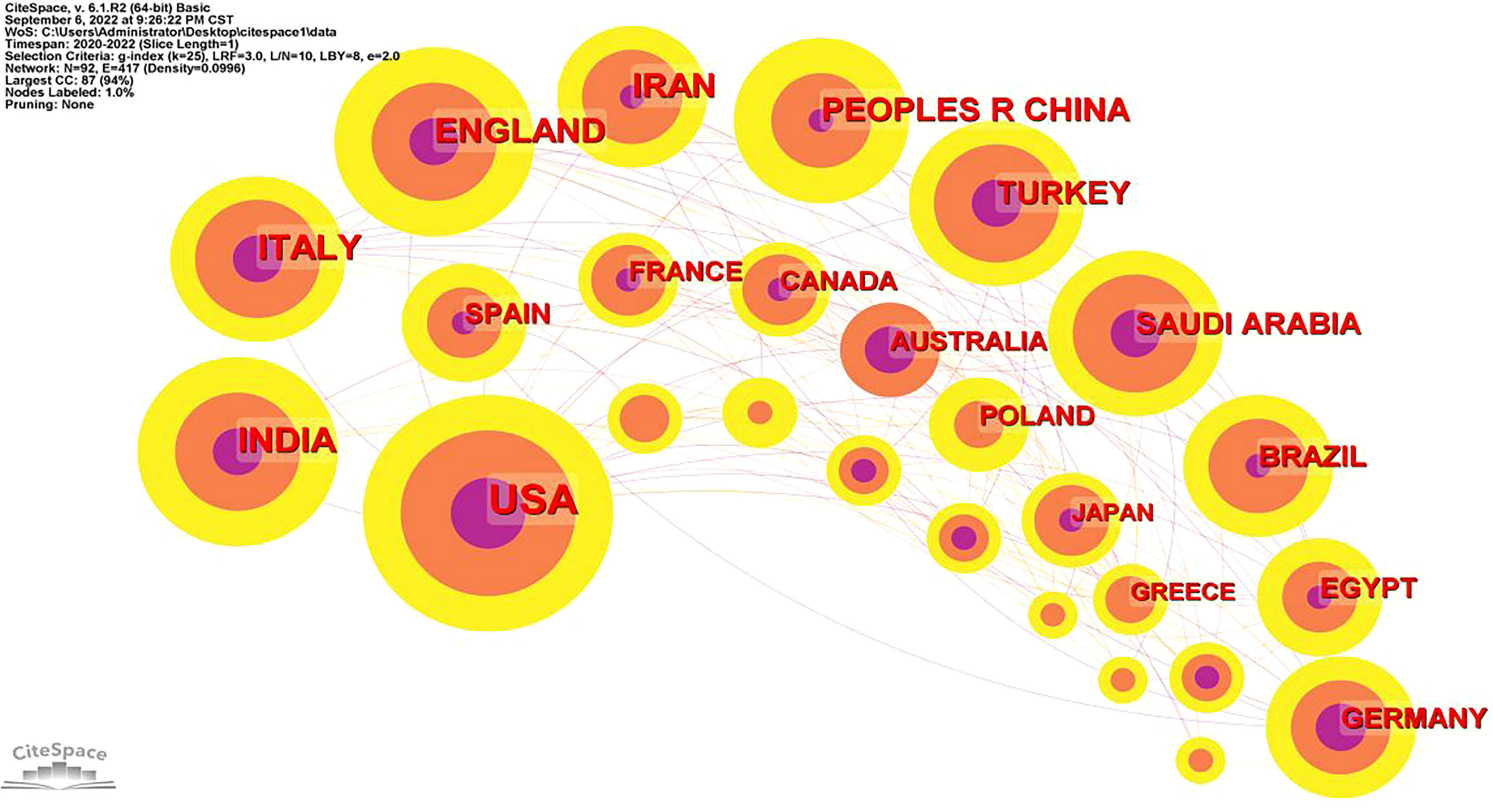
Network of cooperation among countries/regions.

As depicted in **Figure 5**, there are 417 links and 92 nodes in the network of cooperation between countries/regions. The size of each node is proportional to the number of publications, and it represents a country/region. The connection between nodes means cooperation between countries. In the field, 92 countries/regions contributed to this topic. The top 10 countries/regions are listed in **Table 7**. Most publications were published in the United States of America (186,21.75%), followed by The Republic of India (85,9.94%), The Republic of Italy (82,9.59%), The United Kingdom of Great Britain and Northern Ireland (69,8.07%), Islamic Republic of Iran (66,7.72%) and the People’s Republic of China (63,7.37%). The country with the highest degree of centrality was The United States of America (0.55), followed by The United Kingdom of Great Britain and Northern Ireland (0.34), and The Republic of Italy (0.20).

**Table 7:** Top 10 countries/regions with the largest number of articles.

Rank	Country/region	Publications	% (N=855)	Centrality
1	The United States of America	186	21.75	0.55
2	The Republic of India	85	9.94	0.12
3	The Republic of Italy	82	9.59	0.20
4	The United Kingdom of Great Britain and Northern Ireland	69	8.07	0.34
5	Islamic Republic of Iran	66	7.72	0.03
6	the People’s Republic of China	63	7.37	0.03
7	The Republic of Turkey	48	5.61	0.05
8	Kingdom of Saudi Arabia	46	5.38	0.09
9	The Federative Republic of Brazil	37	4.33	0.06
10	The Arab Republic of Egypt	32	3.74	0.07

### Frequency, centrality and cluster analysis of keywords

**Table 8** shows the top 6 keyword co-occurrence frequencies. The word that came up most often was vitamin D (302 times), followed by d deficiency (140 times), infection (105 times), risk (63 times), d supplementation (62 times), and vitamin C (60 times).

**Table 8:** Top 6 frequency and centrality of keywords.

Rank	frequency	Keywords	Centrality	Rank	Centrality	Keywords	Frequency
1	302	vitamin D	0.00	1	0.29	t cell	16
2	140	d deficiency	0.01	2	0.26	randomized trial	8
3	105	infection	0.00	3	0.25	critically ill	5
4	63	risk	0.00	4	0.24	ventilator associated pneumonia	5
5	62	d supplementation	0.10	5	0.22	polyunsaturated fatty acid	9
6	60	vitamin C	0.03	6	0.22	gene	4

**Figure 6:**
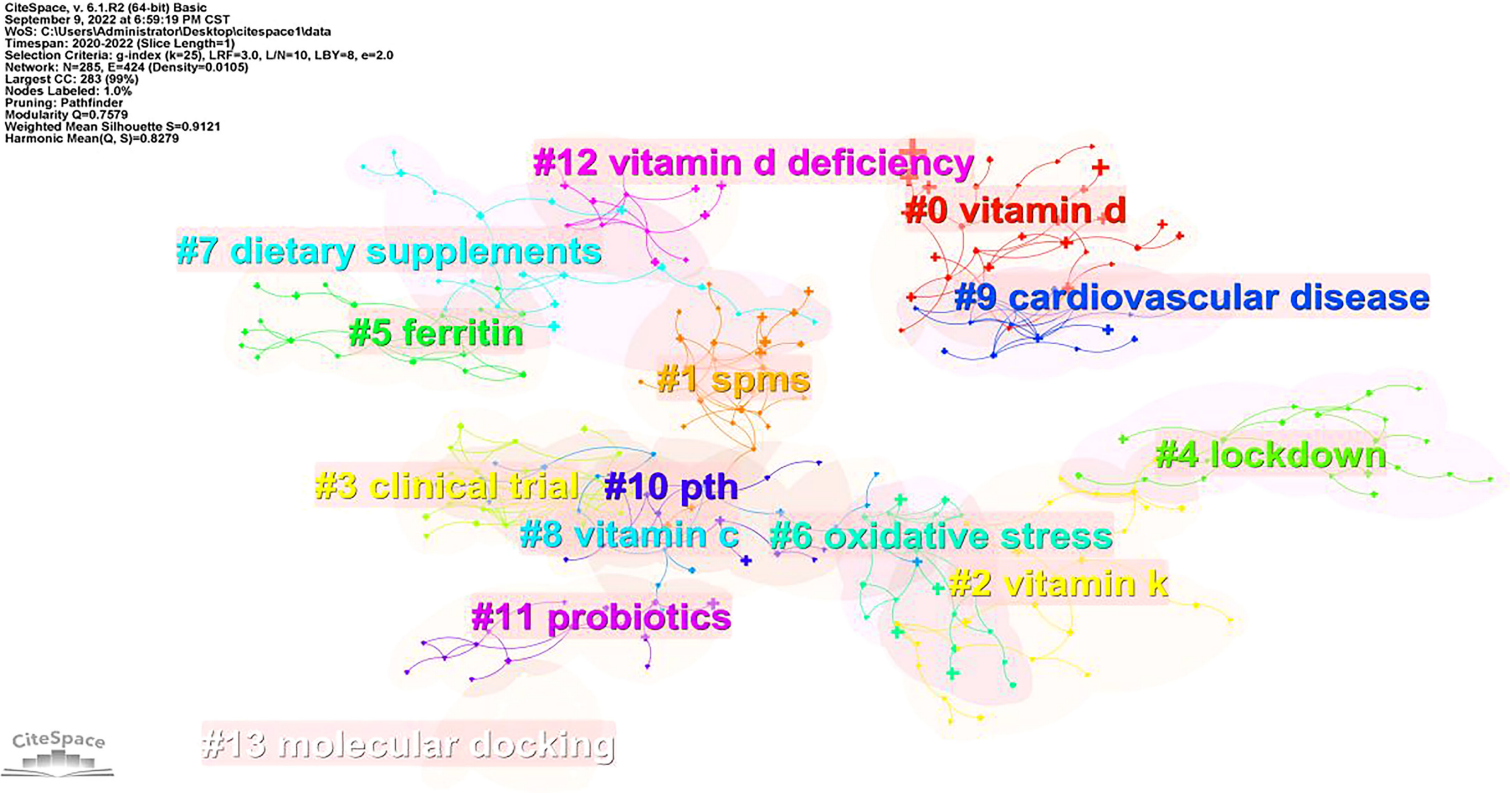
Map of Keyword clusters analysis.

The greater the centrality value, the higher the centrality. The high centrality keywords suggest hotspots in this field, and its value is between 0 and 1. The top 6 keywords, in terms of centrality, were t cell, randomized trial, critically ill, ventilator associated pneumonia, polyunsaturated fatty acid, and gene. A map of the keyword clusters of the COVID-19 and Dietary Supplements is shown in **Figure 6**. Keywords were analyzed with a log-likelihood test cluster analysis based on keywords co-occurrence analysis. In this study, 14 clusters were obtained. See **Figure 6** for details. A significant cluster structure was indicated by the Q value (cluster module value) of 0.7579 (>0.3). Also, the S value, or average profile value, was 0.9121, indicating that the cluster members were extremely consistent.

The top nine keywords clusters were selected for analysis, and listed in **Table 9**. They were “vitamin D”, “Spms”, “vitamin K”, “clinical trial”, “Iockdown”, “ferritin”, “oxidative stress”, “dietary supplements”,and “vitamin C”. Each cluster profile had a value greater than 0.5, indicating a high level of homogeneity and consistency in the clustering.

**Table 9:** Top 9 keywords clusters.

Cluster ID	Clusters Name	Scale	Modularity value	Centrality
#0	vitamin D	30	0.929	vitamin D (66.83,0.0001); cytokine storm (19.9, 0.0001); zinc (13.59, 0.001); probiotics (9.8, 0.005); mortality (9.61, 0.005)
#1	Spms	29	0.814	spms (9.18, 0.005); innate immunity (9.1, 0.005); minerals (6.1, 0.05); infectious disease (6.1, 0.05); 25(oh)d (5.58, 0.05)
#2	vitamin K	27	0.872	vitamin K (16.21, 0.0001); vitamin D (13.92, 0.001); trace element (11.95, 0.001); matrix gla protein (11.44, 0.001); trace elements (11.44, 0.001)
#3	clinical trial	24	0.903	clinical trial (20.22, 0.0001); omega-3 fatty acids (10.93, 0.001); gut microbiome (9.98, 0.005); asymptomatic covid-19 cases (6.72, 0.01); different subtypes of vitamin K (6.72, 0.01)
#4	Iockdown	24	0.990	lockdown (12.04, 0.001); chloroquine (10.06, 0.005); diet (6.7, 0.01); ethnicity (6.7, 0.01); iron homeostasis (6.01, 0.05)
#5	ferritin	22	0.943	ferritin (31.46, 0.0001); iron metabolism (31.46, 0.0001); hepcidin (28.3, 0.0001); lymphocytes (12.32, 0.001); hyperferritinemia (12.32, 0.001)
#6	oxidative stress	21	0.921	vitamin D (14.29, 0.001); oxidative stress (12.66, 0.001); zinc (11.91, 0.001); vitamin C (10.8, 0.005); vitamin a (7.22, 0.01)
#7	dietary supplements	20	0.887	dietary supplements (25.78, 0.0001); rapid review (9.96, 0.005); covid-19 pandemic (8.56, 0.005); zinc (6.78, 0.01); cpg suppression (4.97, 0.05)
#8	vitamin C	20	0.903	vitamin C (23.16, 0.0001); ascorbic acid (19.72, 0.0001); coronavirus disease 2019 (covid-19) (10.61, 0.005); intravenous vitamin C (9.14, 0.005); sepsis (6.35, 0.05)

## Discussion

We used CiteSpace to conduct a comprehensive bibliometric analysis of 855 articles about COVID-19 and dietary supplements.We analyzed the following interesting phenomena.

### Analysis of journals and references

7.13% of articles on “COVID-19 and Dietary Supplements” were published in *Nutrients*. *Nutrients* not only published the largest number of articles on “COVID-19 and Dietary Supplements”, but also ranked first in terms of citation frequency. *Nutrients* is a Q1 journal with a significant international impact and high academic standing. The quality of its research in the direction of nutrition is very high, and publication in a high-quality journal facilitates collaboration and communication among scholars from different countries/regions. Among the top 5 most frequently cited co-cited references, 4 articles discuss vitamin D and respiratory diseases such as acute respiratory infections, influenza, and COVID-19. This suggests that applied research directions for vitamin D are particularly prominent in respiratory infections, especially in COVID-19. Vitamin D is the main dietary supplement for COVID-19. However, whether vitamin D is beneficial for the prevention and treatment of COVID-19, especially how to balance the risks of high-dose use, both deserve further study. The most co-cited article by Adrian R Martineau (2017) said that vitamin D supplementation was safe and effective. 25 eligible randomised controlled trials (11321 participants, aged 0 to 95 years) concluded that vitamin D supplementation reduced the risk of acute respiratory infections in all participants (15). William B Grant (2020) concluded that there was some evidence that vitamin D might reduce the risk of COVID-19. Higher doses of vitamin D3 might be useful for treatment of people infected with COVID-19 (16). However, there are different voices. The relationship between vitamin D and COVID-19 remains controversial. The findings of Claire E Hastie (2020) did not support the potential link between 25 (OH) D concentration and the risk of severe COVID-19 infection and mortality (19). Considering the possible negative effects of high-dose vitamin D3 on bone mineral density (20), more randomized trials may be needed to prove the beneficial effect of vitamin D in preventing severe COVID-19 response or death. In addition, the cross-sectional analysis with the third frequency but the highest centrality found that there was a significant rough relationship between the level of vitamin D and the number of cases of COVID-19, especially the elderly with the most severe vitamin D deficiency (17).

### Analysis of authors, institutions and countries/regions

We found that Wang has made a significant contribution to the field with 10 publications, and Grant’s articles were the most cited (Citations:812 times).Wang’s major studies on COVID-19 and dietary supplements include vitamin D, traditional Chinese medicine and vitamin C. In particular, many studies have been done on whether intravenous vitamin C can benefit COVID-19 patients. Several studies suggested that high doses of intravenous vitamin C may have beneficial effects on inflammatory response, immunity, and deterioration of organ function in COVID-19 patients (21, 22). However, a meta-analysis results showed that short-term intravenous vitamin C treatment could not reduce the severity and mortality of COVID-19 patients (23). According to the ranking of the top ten research institutions of “COVID-19 and Dietary Supplements”, we found that Shahid Beheshti University of Medical Sciences (22 Publications) and Iran University of Medical Sciences (11 Publications), the top two institutions in terms of the number of papers issued, both belong to Islamic Republic of Iran, and the research institutions of Islamic Republic of Iran account for 40% of the top ten. In particular, there is a close communication between domestic research institutions and groups with Shahid Beheshti University of Medical Sciences as the center. There is also a certain degree of exchange and cooperation with institutions in other countries, such as the University of Sao Paulo. Most of the top 10 institutions are universities in various countries. As a result, universities may pay more attention to scholars’ research than other types of research institutions, and also devote significant time, effort and resources to the proper development of research. Among the top 10 research institutions, “Sunlight, Nutrition and Health Research Center” and “Boston University” are from the United States, and their ranking is lower. However, in terms of the total number of publications, most publications were published in the United States (186, 21.75%). It is obvious that the United States has extensive research in this field.

### Research hotspots and trends

By analyzing keywords, our study found that the high-frequency keywords of the current research focus on “COVID-19 and Dietary Supplements” were vitamin D, D deficiency, infection, risk, D supplementation and vitamin C. It showed that vitamin D, vitamin C and COVID-19 infection and risk were the focus of scholars’ attention. However, the centrality is not particularly high, and the keywords of high centrality are T cell, randomized trial, critically ill, inventor, associated pneumonia, polysaturated fat acid and gene. This further indicates that the basic research and clinical trials on “COVID-19 and Dietary Supplements” are being actively carried out, such as the impact on t cells, polysaturated fat acid and gene, or the research on randomized trials such as ventilator associated pneumonia and critically ill. By analyzing keywords clusters, our study found that several hot aspects of “COVID-19 and Dietary Supplements” research are dietary supplements (vitamin D, vitamin K, vitamin C), mechanisms (ferritin, specialized pro-resolving mediators (SPMs), oxidative stress), research methods (clinical trials), and the typical prevention and treatment strategies (lockdown) of COVID-19. The lockdown is a public health strategy for the prevention and treatment of COVID-19. We hypothesized that this strategy might affect dietary supplementation by affecting people’s lifestyles. However, data on the relationship between lockdown measures and human nutrients such as vitamin D, vitamin K, vitamin C are scarce and warrant further exploration. Vitamin D is the focus of current research. Studies have confirmed that vitamin D plays a protective role in various fields of oncology (24), neurology (25, 26) and immunology (27) in addition to the well-known effects on bones (28).

By exposing themselves to the beta component of UV sunlight, humans naturally produce vitamin D. Vitamin D levels produced naturally are sufficient to meet the fundamental requirements of bone metabolism (29).Today, due to the COVID-19 pandemic, lockdown measures have resulted in more people needing vitamin D3 (cholecalciferol) supplementation due to lack of exercise, especially sunbathing. Older people need vitamin D supplementation because of their higher risk of osteoporosis. Compared with patients with severe COVID-19, patients with non-severe COVID-19 had higher levels of calcitriol (30, 31). Recent meta-analyses and systematic reviews have shown that taking vitamin D supplements as part of a healthy diet reduced the risk of COVID-19-related severe disease and death (32, 33), whereas another study did not support the role of vitamin D in the progression and outcome of COVID-19 (34).

### Vitamin D and COVID-19

Vitamin D is the main dietary supplement for COVID-19. However, whether vitamin D is beneficial for the prevention and treatment of COVID-19, especially how to balance the risks of high-dose use, both deserve further study. The most co-cited article by Adrian R Martineau (2017) said that vitamin D supplementation was safe and effective. 25 eligible randomised controlled trials (11321 participants, aged 0 to 95 years) concluded that vitamin D supplementation reduced the risk of acute respiratory infections in all participants (15). William B Grant (2020) concluded that there was some evidence that vitamin D might reduce the risk of COVID-19. Higher doses of vitamin D3 might be useful for treatment of people infected with COVID-19 (16). However, there are different voices. The relationship between vitamin D and COVID-19 remains controversial. The findings of Claire E Hastie (2020) did not support the potential link between 25 (OH) D concentration and the risk of severe COVID-19 infection and mortality (19). Considering the possible negative effects of high-dose vitamin D3 on bone mineral density (20), more randomized trials may be needed to prove the beneficial effect of vitamin D in preventing severe COVID-19 response or death. In addition, the cross-sectional analysis with the third frequency but the highest centrality found that there was a significant rough relationship between the level of vitamin D and the number of cases of COVID-19, especially the elderly with the most severe vitamin D deficiency (17).

## Conclusion

There are no guidelines on dietary supplements for the prevention or treatment of COVID-19, while basic research and clinical trials on “COVID-19 and Dietary Supplements” are still popular. After all, dietary supplements are inextricably linked to people’s lives. We have summarized and analyzed these studies, and found that vitamin D, vitamin K, and vitamin C are the main research focuses at present. Among them, vitamin D is the main dietary supplement for COVID-19. However, whether vitamin D is beneficial for the prevention and treatment of COVID-19, especially how to balance the risks of high-dose use, both deserve further study. In terms of mechanism of action, ferritin, specific slow-release mediators (SPMs) and oxidative stress are worth further investigated. Besides, the lockdown measures have already changed people's lives. It is worth pondering whether such changes affect the balance of nutrients in the body. Whether intake of dietary supplements under lockdown measures can help prevent COVID-19 deserves further research. As one of the natural nutrients, it is worthy of further study on the effectiveness and safety of herbs during the COVID-19 pandemic.

## Limitations

This study inevitably has some limitations that need to be refined in the future. There are various databases such as Pubmed, Cochrane Library and Scopes. We only searched articles and review articles written in English through the WOS core database, which may lead to potentially incomplete data.Only articles from 2019 to September 3, 2022, were selected, articles published after that date were not included in this study. Therefore, if this study was repeated by researchers under other conditions, the results could be different. CiteSpace software was used for analysis in this work. However, minor errors due to database or software problems may still occur.

## Data availability statement

The raw data supporting the conclusions of this article will be made available by the authors, without undue reservation.

## Author contributions

Data curation, WH,YX. Formal analysis,WH. Supervision, YX. Writing – original draft, WH and YX. Writing – review & editing, WH and YX. All authors contributed to the article and approved the submitted version.

## Conflict of interest

The authors declare that the research was conducted in the absence of any commercial or financial relationships that could be construed as a potential conflict of interest.

## Publisher’s note

All claims expressed in this article are solely those of the authors and do not necessarily represent those of their affiliated organizations, or those of the publisher, the editors and the reviewers. Any product that may be evaluated in this article, or claim that may be made by its manufacturer, is not guaranteed or endorsed by the publisher.
